# Supplementation of omega-3 and dietary factors can influence the cholesterolemia and triglyceridemia in hyperlipidemic Schnauzer dogs: A preliminary report

**DOI:** 10.1371/journal.pone.0258058

**Published:** 2021-10-19

**Authors:** Paula de Albuquerque, Viviani De Marco, Thiago Henrique Annibale Vendramini, Andressa Rodrigues Amaral, Sergio Catanozi, Kelly Gomes Santana, Valéria Sutti Nunes, Edna Regina Nakandakare, Marcio Antonio Brunetto

**Affiliations:** 1 Universidade de Santo Amaro, São Paulo, Brazil; 2 Faculdade de Medicina Veterinária e Zootecnia, Centro de Pesquisa em Nutrologia de Cães e Gatos, Universidade de São Paulo, Sao Paulo, SP, Brazil; 3 Faculdade de Medicina, Laboratorio de Lipides (LIM—10), Hospital das Clinicas HCFMUSP, Universidade de São Paulo, Sao Paulo, SP, Brazil; 4 Naya Especialidades, São Paulo, SP, Brazil; University of Illinois, UNITED STATES

## Abstract

Primary hyperlipidaemia in Schnauzer is characterized by increased plasma triglycerides (TG) and/or total cholesterol (TC) concentration and is associated with an increased risk of developing pancreatitis, insulin resistance and seizures. In humans, omega-3 fatty acids in addition to a low-fat diet can be used to reduce TG and TC. This study evaluated the therapeutic efficacy of omega-3 fatty acids associated to a diet management with two different fat content in Schnauzer with primary hyperlipidaemia. Eighteen dogs with primary hyperlipidaemia were divided into two groups: group 1, n = 10, 8 females, 2 males, age (mean ± standard deviation) of 7.13 ± 2.70 years and body weight (BW) (mean ± standard deviation) of 7.25 ± 1.22 kg were treated with fish oil (approximately 730 mg/day of omega-3) associated with a low-fat and low-calorie diet (approximately 24g of fat/1000 kcal) for 90 days (T90); and group 2, n = 8 dogs, 6 females, 2 males, with 7.0 ± 1.77 years old and average BW of 8.36 ± 1.51 kg, treated with fish oil (approximately 730 mg/day of omega-3) and maintenance diet with moderate amount of fat (approximately 33g of fat/1000 kcal) for 90 days. Plasma TG and TC concentrations and lipoprotein (LP) profile (VLDL, LDL, HDL) were evaluated before and after treatment. TG and TC serum concentrations, expressed in mg/dL (mean ± standard deviation), before and after treatment in group 1 were: TG = 391.30 ± 487.86 (T0) and 118.7 ± 135.21 (T90); TC = 308.2 ± 63.06 (T0) and 139 ± 36.91 (T90). As for group 2, TG = 391.63 ± 336.89 (T0) and 250.75 ± 211.56 (T90); TC = 257.25 ± 92.88 (T0) and 207.25 ± 63.79 (T90). A reduction (p<0.05) of TG and TC was observed in both groups. The distribution of TG and TC among LP was not different between the pre (T0) and post treatment (T90) periods. After 90 days of treatment, the administration of omega-3 fatty acids, associated with a low-fat or maintenance diet reduced triglyceridemia and cholesterolemia without altering LP profile. The current investigation shows that both therapies were effective in reducing plasma TC and TG concentrations without altering LP profile.

## Introduction

The increase in blood lipid concentrations is called hyperlipidaemia. This increase can be caused by total cholesterol (TC) (hypercholesterolaemia), triglycerides (TG) (hypertriglyceridaemia) or both (mixed hyperlipidemia) [1,2]. On the other hand, dyslipidemia refers to any type of disorder that changes the characteristics of lipids and lipoproteins (LP) and not only to the increase in blood lipid concentrations [3]. There are 3 types of hyperlipidemia: physiological (postprandial), secondary (to diseases or medications) and primary (familial/hereditary) [2]. Postprandial hyperlipidemia is physiological and transient and resolves in 7 to 12 hours after a meal and is dependent on the amount of fat consumed [3–5]. For this reason, determination of serum lipid concentration should be performed with a 12-hour fast, however, Xenoulis and Steiner [3] implements a 15-hour fast before evaluating dogs for hyperlipidemia.

Secondary hyperlipidaemia is the most common form in dogs, and it can be a result of endocrine disorders (e.g. diabetes mellitus, hypothyroidism, hyperadrenocorticism), pancreatitis, cholestasis, protein-losing nephropathy, obesity, high-fat diets, as well as the use of certain drugs (e.g. glucocorticoids, phenobarbital, potassium bromide) [1,6,7]. On the other hand, primary hyperlipidaemia is less common in the general canine population but it can be very common within certain breeds as Schnauzers.

Primary hyperlipidemia is very common in miniature Schnauzers and it is related to hereditary factors [2,8,9]. The etiology of Schnauzer’s primary hyperlipidemia has not been well established but it may be caused by increased production or reduced clearance of very low density LP (VLDL) and chylomicrons (CM) or both [1,3,5,8]. Primary hyperlipidemia has also been described in other breeds such as Shetland Shepherd, Bull Terrier, Briads, Rough Collie, Doberman and Rottweillers, although in these breeds, hypercholesterolaemia is more prevalant. There is also a report that Beagles may present familial hypertriglyceridaemia [1,2,8] as well. The first step in treatment of hyperlipidemia is to determine whether it is primary or secondary [3,10,11] and, therefore, tests should be performed to rule out all the differential diagnoses that could cause secondary hyperlipidemia.

Modifying the diet is the first step of primary hyperlipidemia therapy [3,12]. A recent study by Xenoulis et al. (2020) demonstrated that the low-fat diet was significantly effective in reducing both cholesterol and triglycerides, when used for a period of 2 months, in hyperlipidemic schnauzers. In addition to the low-fat diet, omega-3 fatty acids have been used. There are reports in humans that described their benefits in reducing TG and TC [1,3,10,13,14]. However, there are no clinical studies in primary hyperlipidemic dogs to date, which have evaluated the hypolipidemic effect of omega-3, although in clinical practice it is widely used by veterinarians.

Thus, this study aimed to evaluate the therapeutic efficacy of omega-3 fatty acids in Schnauzers with primary hyperlipidaemia associated to a low-fat diet in a group and a maintenance diet with moderate fat content in another group by comparing serum TG and TC concentrations and the profile of plasma LP (VLDL, low density LP–LDL—and high density LP—HDL) before and after 90 days of treatment, in both groups.

## Materials and methods

The experimental protocol was conducted in accordance with the ethical principles of animal experimentation and approved by the Ethics Committee on the use of animals at the Santo Amaro University (No. 02/2016). All owners of the participating animals gave written consent.

### Animals and experimental design

Blood samples were collected from 122 miniature Schnauzer dogs from kennels in the city of São Paulo and from the routine of a private veterinary practice (NAYA Especialidades, São Paulo, Brazil), from July 2015 to February 2017.

Among the 122 dogs, 20 presented primary hyperlipidemia (triglycerides >150mg/dL and/or cholesterol >300mg/dL) [15] and were included in the study. Thus, animals with hypercholesterolemia, hypertriglyceridemia, or mixed hyperlipidemia (animals that have both) were included. The animals were randomly assigned to two experimental groups. During the study, two animals from Group 2 were excluded, one because their owners did not comply with the experimental protocol and the other due to the development of a cortisol-secreting tumor in the adrenal gland.

The therapeutic protocol for the experimental groups was as following: Group 1 (low-fat diet + omega-3) - 10 dogs; 8 females, 2 males, age (mean ± standard deviation) of 7.13 ± 2.70 years and body weight (BW) (mean ± standard deviation) of 7.25 ± 1.22 kg given 1 capsule of fish oil (approximately 730 mg/day of omega-3; Ograx 3, Avert^®^, São Paulo, Brazil) and low calorie diet (Equilíbrio Veterinary O&D, Total Alimentos^®^, Três Corações, Brazil) with low fat content (approximately 24g of fat/1000 kcal) for 90 days; and Group 2 (moderate-fat diet + omega-3) - 8 dogs; 6 females, 2 males, age of 7.0 ± 1.77 years and average BW of 8.36 ± 1.51 kg given 1 capsule of fish oil (approximately 730 mg/day of omega-3; Ograx 3, Avert^®^, São Paulo, Brazil) and maintenance diet (Golden Formula^®^, Grandfood Industry, Dourado, Brazil) with moderate amount of fat (approximately 33g of fat/1000 kcal) for 90 days.

Omega-3 supplementation was made through fish oil jelly capsules, composed by 412mg of EPA and 318.1mg of DHA in the proportion of 1.5:1 respectively, administered to the animals once a day at the time of the meal. This dose was equivalent to 58.8mg/kg of BW of EPA and 45.4mg/kg of BW of DHA.

The composition of the maintenance and hypocaloric diets as well as the composition of the fish oil capsules are described in Tables 1 and 2, respectively.

**Table 1:** Composition of the diets used in the study.

Item	Moderate fat diet	Low-fat diet
	unit/kg	unit/kg
Moisture	79.6g	85.4g
Crude protein	255.1g	305.3g
Fat	126.0g	74.00g
Ash	53.0g	46.2g
Crude fiber	19.1g	22.2g
Calcium	11.3g	11.7g
Phosphorus	8.5g	9.0g
Metabolizable energy	3795kcal	3100kcal

**Table 2:** Composition of the fish oil capsules used in the study.

Item	unit/kg
Fat	999.4 g
Polyunsaturated fats	801.6 g
Monounsaturated fats	135.5 g
Unsaturated fats	937.0 g
Saturated fat	62.0 g
Trans fat	0.9 g
Myristic acid (C14:0)	0.6 g
Pentadecanoic acid (C15:0)	0.1 g
Palmitic acid (C16:0)	18.4 g
Margharic acid (C17:0)	2.1 g
Stearic acid (C18:0)	35.8 g
Arachidic acid (C20:0)	5.2 g
Behenic acid (C22:0)	1.4 g
Palmitoleic acid (C16:1)	6.4 mg
Oleic acid (C18:1n9c)	100.8 g
Cis-eicosenoic acid (C20:1)	23.9 g
Erucic acid (C22:1n9)	3.9 g
Nervonic acid (C24:1)	1.0 g
Linoleic acid (C18:2n6c)	20.2 g
Gamma linolenic acid (C18: 3n6)	2.4 g
Linolenic acid (C18:3n3)	9.7 g
Cis-eicosadienic acid (C20:2)	2.1 g
Cis-eicosatrienoic acid (C20:3n3)	1.9 g
Arachidonic acid (C20:4n6)	31.1 g
Cis-docosahexaenoic acid DHA (C22:6n3)	318.1 g
Cis-eicosapentaenoic acid EPA (C20:5n3)	412.0 g
Elaidic acid (C18:1n9t)	0.9 g

The amount of food fed was determined according to the recommendations of energy requirement for BW maintenance in adult dogs: 95 x (body weight)^0.75^ [16,17]. The food was provided by the owners of the dogs and it was divided into two daily meals. None of the foods had EPA and DHA in their composition.

The diagnosis of hyperlipidemia was based on serum TC concentrations greater than 300mg/dL and TG greater than 150mg/dL [15] after a 12-hour fast.

The inclusion criteria for this experiment were: absence of clinical symptoms for any concurrent diseases; absence of a history of chronic diseases that could cause hyperlipidemia, such as hypothyroidism, diabetes mellitus, hyperadrenocorticism, pancreatitis, cholestasis or nephrotic syndrome; serum concentrations of free T4 within the reference range for the species in order to exclude hypothyroidism; normal serum concentrations of alanine aminotransferase (ALT) and alkaline phosphatase (ALP) species; body condition score between 4 and 6 according to the 9-point scale proposed by Laflamme [18]. The exclusion criteria were: use of drugs that could increase TG and TC plasma concentrations, such as glucocorticoids and phenobarbital, up to three months before the study; use of drugs that could reduce TG and TC plasma concentrations, such as fibrates, statins, ursodesoxycholic acid and omega-3; dogs previously fed a homemade, hypocaloric or lipid-lowering diets.

All dogs were submitted to anamnesis, physical examination and laboratory tests previously the beginning therapy (T0) and 90 days after treatment (T90).

### Blood collection, sample processing and analysis

After an 12-hour fast, blood samples were aseptically collected from the jugular vein with a syringe and then the sample was distributed as follows: 3mL in test tubes for dry biochemistry (red tube) and 2mL for test tube with the EDTA anticoagulant.

Blood samples were kept on ice and taken to laboratories within 2 hours in order to be centrifuged and immediately analyzed (TG, TC, glucose, ALT, ALP, Free T4) or frozen -20°C for later analysis (lipoproteins—LP). In samples destined for the dosage of LPs, a preservative was added before freezing, composed of 2mM Benzamidine (Sigma B-6506, Sigma-Aldrich Corporation, Missouri, United States); 0.5% Gentamicin; 0.25% Chloramphenicol; 2mM Aprotinin (Sigma A-6270, Sigma-Aldrich Corporation, Missouri, United States) e 0.5mM pMSF (Sigma P-7626, Sigma-Aldrich Corporation, Missouri, United States).

Biochemical analyzes were performed at the Clinical Analysis Laboratory of NAYA specialties (São Paulo, Brazil), with the semi-automatic biochemical analyzer BIO-200® (Bioplus, Barueri, Brazil). Serum concentrations of ALT, ALP, glucose, TC and TG were determined with commercial kits from Labtest (Santa Bárbara d’Oeste, Brazil); ALT was determined by the UV-IFCC kinetic method, ALP by the modified Bowers and Mc Comb kinetic method, glucose by the GOD-Trinder method, and TC and TG by the colorimetric methods.

Serum concentration of free T4 was performed in the hormonal analysis laboratory of Provet (São Paulo, Brazil), for this purpose, a commercial kit from the company IVD Technologies (California, United States) was used in an Automatic Gamma Counter (Perkin Elmer, São Paulo, Brazil). Free T4 concentration was determined by liquid phase radioimmunoassay with prior preparation of the sample by extraction technique using equilibrium dialysis.

The profile of plasma LP (VLDL, LDL, HDL) was determined at the Lipids Laboratory of the Faculty of Medicine of the University of São Paulo (São Paulo, Brazil) by Fast Protein Liquid Chromatography (FPLC) in AKTA Purifier system (GE Healthcare Bio-Sciences, Uppsala, Sweden). Plasma aliquots (100μL) from each animal were injected into a Superose 6HR 10/30 column (GE Healthcare 17-5172-01). The plasma was eluted, in a constant flow of 0.5mL/min, with Tris buffer (10nM Tris, 150mM NaCl, 1mM EDTA and 0.03% NaN3), pH 7.4. Fractions were collected and pooled according to the elution times expected for VLDL, LDL, and HDL. TC and TG concentrations were measured in aliquots of isolated lipoprotein fractions [19].

### Statistical analysis

Statistical analyzes were performed with the GraphPad Prism Version 5.0® software. Comparisons between variables before and after treatment in both groups were performed using the paired Student’s T test (mean ± SD); p-value <0.05 was considered significant.

## Results

Blood samples were collected from 122 healthy Schnauzers. Among these, 20 (16.4%) had plasma TG and/or TC concentrations above the upper limit of the reference range. Two dogs were excluded from the study, and the final sample included 18 Schnauzers (14.75%) with primary hyperlipidaemia, which were 14 females (6 entire and 8 neutered) and 4 neutered male (Table 3).

**Table 3:** Hyperlipidemic Schnauzer’s profile.

Animal	Age (years)	Sex	Body weight (Kg)	BCS	Reproductive status	Diagnosis
*Group 1 (low-fat diet + omega-3)*						
1	4	F	6.5	5	Non-castrated	HTC
2	1	F	6.1	5	Non-castrated	HTC
3	1	F	5.6	5	Non-castrated	HTC and HTG
4	12	M	6.9	5	Castrated	HTC
5	7	F	7.0	6	Castrated	HTG
6	5	F	7.2	6	Non-castrated	HTC and HTG
7	5	F	6.6	5	Non-castrated	HTC and HTG
8	6	F	8.5	6	Castrated	HTG
9	9	M	9.2	6	Castrated	HTG
10	9	F	8.9	6	Castrated	HTC and HTG
*Group 2 (moderate fat diet+ omega-3*)						
11	8	F	8.7	6	Castrated	HTC and HTG
12	8	M	9.0	6	Castrated	HTG
13	7	F	8.3	6	Castrated	HTG
14	8	F	9.6	6	Castrated	HTC and HTG
15	8	M	9.0	6	Castrated	HTG
16	8	F	7.0	5	Castrated	HTG
17	3	F	7.5	6	Non-castrated	HTC
18	6	F	8.0	6	Castrated	HTG
**Average**	**6.39**	**..**	**7.97**	**5.67**	**..**	**..**

BCS = body condition score (Laflamme, 1997); HTC = hypercholesterolaemia; HTG = hypertriglyceridaemia.

Eight dogs (n = 8/18; 45%) had only hypertriglyceridaemia and 4 (n = 4/18; 22%) only hypercholesterolaemia. Six dogs (n = 6/18; 33%) presented mixed hyperlipidaemia (ie, increased TG an TC plasma concentrations).

Regarding the type of hyperlipidemia, 4 animals in Group 1 (n = 4/10; 40%) presented mixed hyperlipidaemia, 3 (n = 3/10; 30%) hypertriglyceridaemia and 3 (n = 3/10; 30%) only hypercholesterolaemia. In Group 2, 5 dogs had (n = 5/8; 62.5%) hypertriglyceridaemia, 2 animals (n = 2/8; 25%) had mixed hyperlipidaemia and 1 hypercholesterolaemia (n = 1/8; 12.5%).

**Fig 1:**
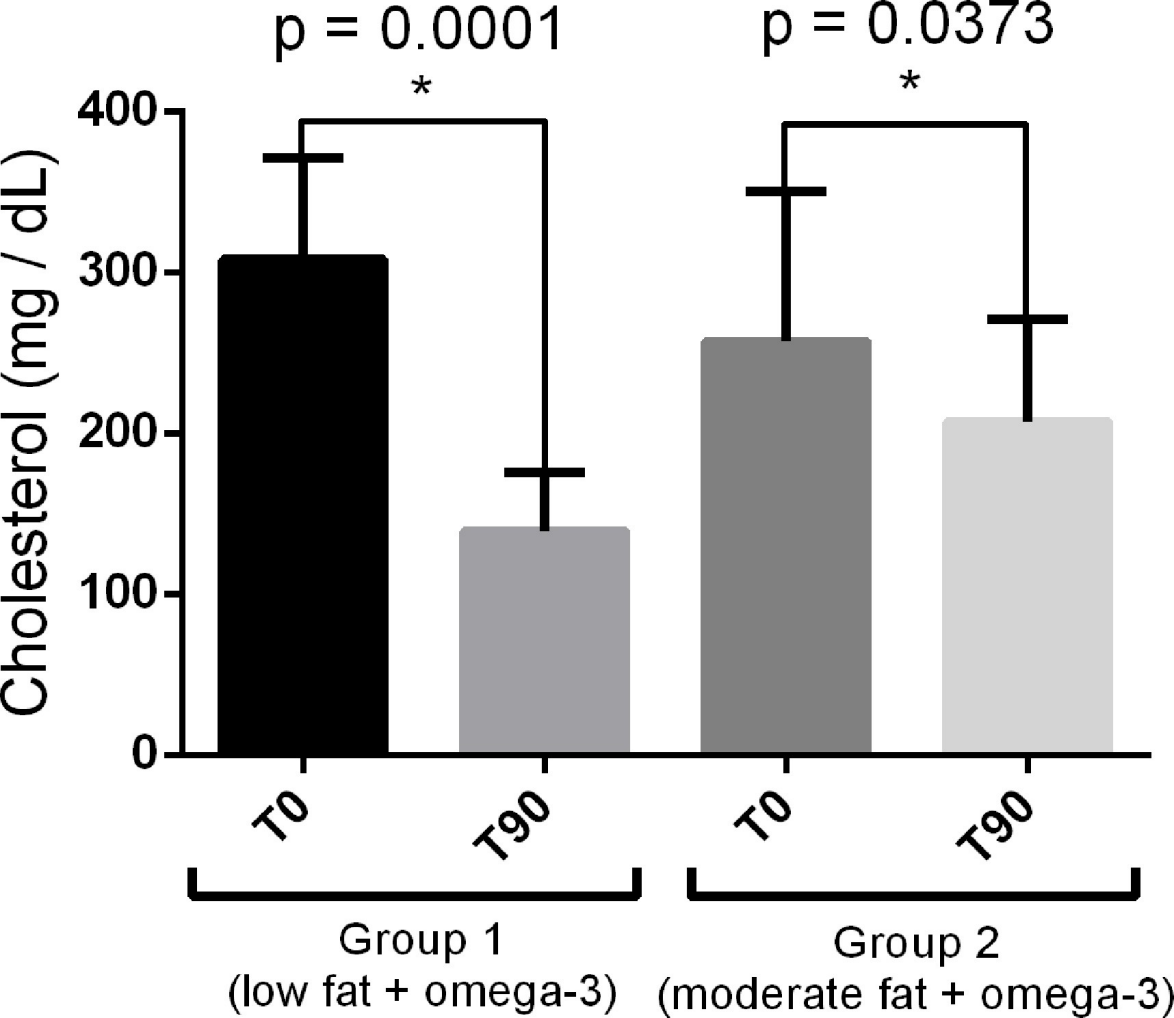
Serum cholesterol concentrations in the experimental groups before (T0) and 90 days after treatment (T90).

**Fig 2:**
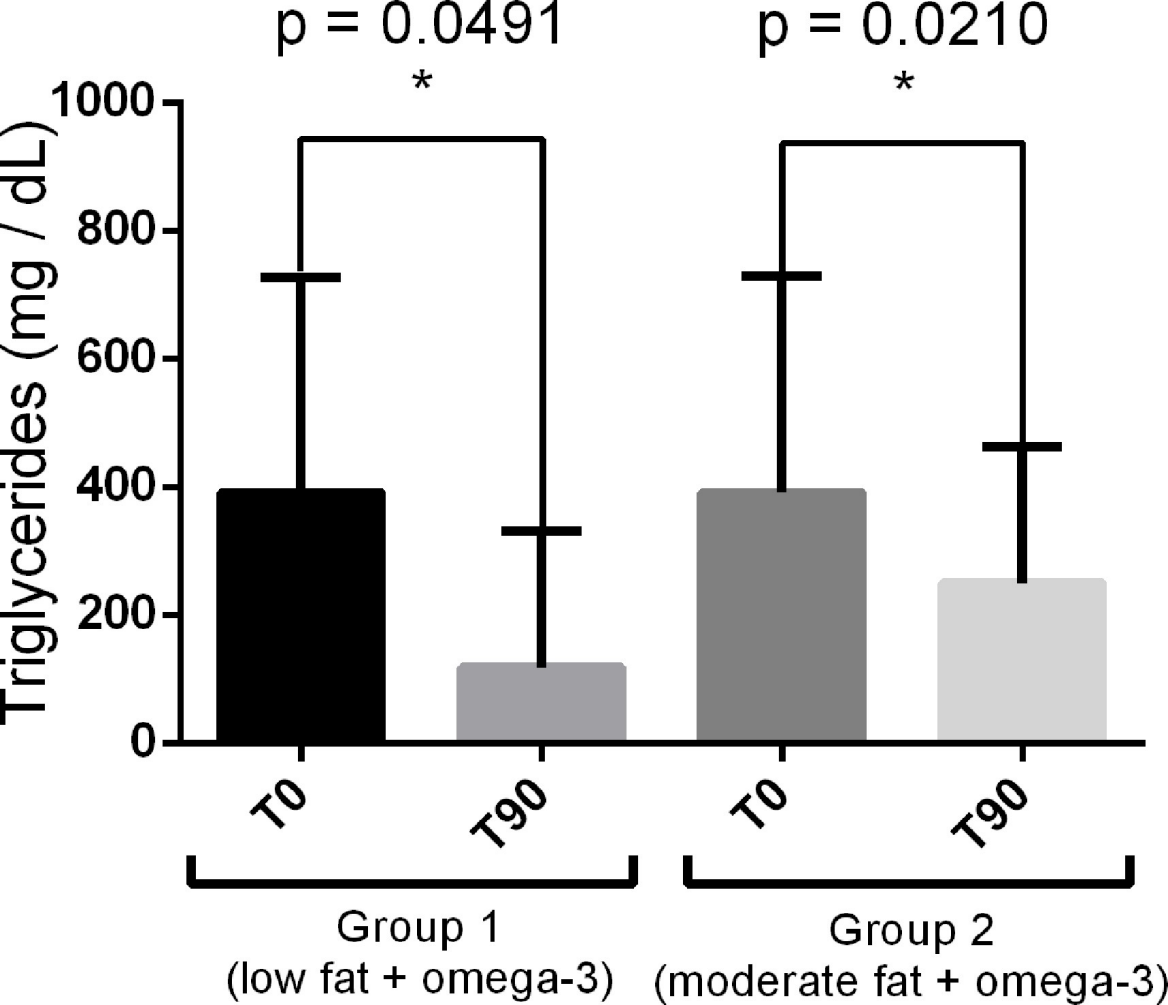
Serum triglyceride concentrations in the experimental groups before (T0) and 90 days after treatment (T90).

**Fig 3:**
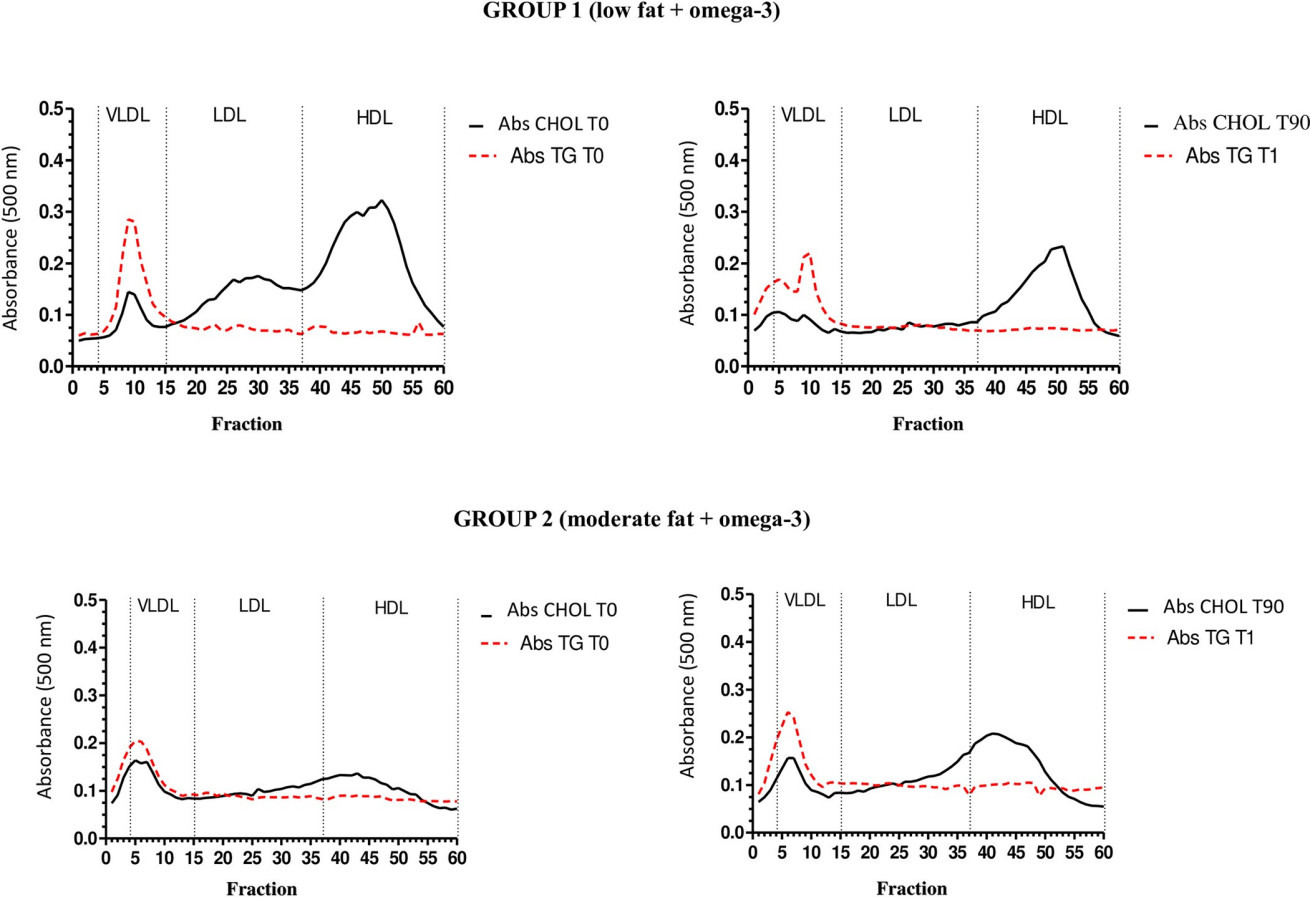
Distribution of lipoprotein fractions in the experimental groups at T0 and T90.

After 90 days of treatment, therefore, the dogs from both groups had a reduction in cholesterolemia and triglyceridemia expressed (mean ± standard deviation). For group 1 (low fat and omega-3), the mean cholesterol values were 308.2 ± 63.06 at T0 and 139 ± 36.91 at T90 (p = 0.0001); for group 2 (moderate fat and omega-3), the respective mean values were 257.25 ± 92.88 at T0 and 207.25 ± 63.79 at T90 (p = 0.0373, Fig 1). Regarding triglyceridemia for group 1, the results found were 391.63 ± 336.89 at T0 and 250.75 ± 211.56 at T90 (p = 0.0491), and in group 2, 391.63 ± 336.89 at T0 and 250.75 ± 211.56 at T90 (p = 0.0210, Fig 2). For the percentual distribution of such lipids among the LP fractions, no differences were observed (Fig 3).

## Discussion

Primary hyperlipidemia was identified in 15% of the investigated healthy Schnauzers and both protocols of therapies were effective in reducing plasma TC and TG concentrations, especially when omega-3 and low-calorie diet were associated.

Xenoulis et al. [20] reported that hyperlipidemia is a common finding in the United States, where 32.8% of the 192 Schnauzers assessed in the study had hyperlipidaemia after a 12-hour fast. In addition, corroborating the findings of Xenoulis et al. [8] and Mori et al. [9], isolated hypertriglyceridaemia (44.45% of cases) and mixed hyperlipidaemia (33.33% of cases) were the most prevalent forms of hyperlipidemia observed in this study. However, isolated hypercholesterolaemia was also observed in 22.22% of cases, a result not yet reported by other authors in Schnauzer dogs.

Whitney et al. [21] and Xenoulis et al. [15] stated that hypercholesterolaemia, when present in Schnauzers, has always been associated with hypertriglyceridaemia. Hypercholesterolaemia associated with hypertriglyceridaemia has been reported in Shetland’s Shepherd [9,22] and Beagles [23]. Isolated hypercholesterolaemia has been reported in Briards and in a Collie family over the UK [24,25], but not in Schnauzers so far. Thus, the etiology of higher plasma TC concentration observed in 4 animals (the other 14 animals had only hypertriglyceridemia) of the current study needs to be further investigated (Table 3).

The cause of Schnauzer’s idiopathic hyperlipidemia is still unknown, but it is suggested that it is hereditary because it is prevalent in a specific breed. Possible mechanisms include increased production or reduced clearance of VLDL and chylomicrons or both [8,9]. Similarly, there is a familial hypertriglyceridaemia in humans, characterized by an increase in the production of VLDL [21,26] and apolipoprotein C-II (APOC2) deficiency, in humans is a very rare cause of hyperkylomycinemia, the most common cause is deficiency of lipoprotein lipase (LPL) [27].

It was suggested that Schnauzer hyperlipidemia could be due to APOC2 deficiency, however Xenoulis et al. [27] have not found coding variants in the APOC2 gene and concluded that APOC2 hereditary deficiency is unlikely to be a major cause for primary hyperlipidemia in this breed.

According to previous investigations, hyperlipidemia is prevalent in dogs older than 6 years old [8,19], however, in the current study, hyperlipidemia was also observed in younger adult dogs (1 year; n = 2), 3 (n = 1), 4 (n = 1), 5 (n = 2), 6 (n = 2) and ≥ 7 (n = 10)-year-old dogs. Mori et al. [9] also reported a higher prevalence of hyperlipidemia in older animals, and concluded that the risk of hyperlipidemia increases with age and is more prevalent in elderly animals.

Of the 18 animals with hyperlipidemia, 14 were females and 4 were males, so that in this study the highest prevalence occurred in females, as reported by Xenoulis et al. [8] and Mori et al. [9] as well. However, it is not yet possible to characterize idiopathic hyperlipidemia as a sex-related disorder. It is also important to note that 45% of the animals came from kennels, where there is a higher frequency of females than males.

Hyperlipidaemia was more prevalent in castrated animals (70%) of this study. Schmidt et al. [28] suggested that plasma TG concentrations in hysterectomized females tend to increase, however Xenoulis et al. [8] did not as a predisposing factor for idiopathic hyperlipidaemia.

In addition, 8/18 (44.45%) of the animals were female, neutered and overweight, with a body condition score that ranged between 5/9 and 6/9 [18]. Brunetto et al. [29] and Vendramini et al. [30] stated is a risk factor for the development of obesity, as it reduces the basal metabolic rate, increases appetite, and may also cause replacement of muscle mass by adipose tissue as a result of the lower concentration of androgenic hormones. Currently, females tend to be early and this could lead to obesity and favor the development of hyperlipidemia [3].

As expected, the low-fat diet + omega-3 (Group 1) was more effective in reducing hyperlipidemia than the moderate-fat diet + omega-3 (Group 2).

Dietary intervention is the most effective practice in the treatment of hyperlipidemia [5]. The carbohydrate source interferes with the digestibility of the diet [28]; and consequently on postprandial glycemic curves in dogs [29,30] and effect on lipid profile of hyperlipidaemic dogs. Recently, the inclusion of peas and barley, as exclusive sources of starch, in therapeutic diets for diabetic dogs can minimize the plasma concentration of triglycerides and cholesterol in fasting and at different postprandial times, compared to the corn diet or diet with lower fat content [31], the diets evaluated in our study had the same starch and protein sources, which we believe did not interfere with the results.

A low**-**fat, high fiber and low-calorie diet decreases the influx of CM and, consequently, TG into the plasma circulation; in addition, the low-fat diet reduces plasma TC concentration and saturated fat sent to the liver [32]. In our study, the low-fat diet + omega-3 (Group 1) also had more fiber (about 16% higher) compared to the other treatment. Dietary fibers act on glucose absorption, improve insulin receptor activity [33], act on the intestinal reabsorption of bile acids so that they can be used for cholesterol synthesis in the liver [33,34].

A low-fat, high-fiber, and calorie-restricted diet decreases the influx of chylomicrons and consequently of triglycerides into the circulation and reduces LPL expression. In addition, a low-fat diet also helps to reduce concentrations of cholesterol and saturated fat sent to the liver [32].

Although the lipid-lowering effects of omega-3 are well established in humans, the mechanisms of action are still not well understood. The lipid-lowering effects of omega-3 can be attributed to the regulation of transcription factors related to lipogenesis; increased β-oxidation; stimulating the activity of the lipoprotein lipase enzyme; decreased cholesterol absorption and reduced concentration of non-esterified fatty acids; decreased intestinal absorption of glucose and lipids; and finally, increased cholesterol secretion through bile salts [3,35–38].

However, although interesting, the low number of dogs enrolled in our study does not allow us to take any real considerations about the prevalence of hyperlipidemia in castrated animals, the relationship with obesity, and the regulation of omega-3 lipid-lowering factors. Another fact to be highlighted is that the results obtained are interesting but reflect the supplementation of omega-3 and dietary factors, without the isolation of the responsible factors being possible.

For humans, studies have indicated an average intake of 100mg of omega-3 per day. Some health organizations recommend 1000mg per week for the prevention of chronic diseases, between 250 and 1000mg per day specifically for the prevention of coronary heart disease and between 2 and 4g per day for the control of hypertriglyceridaemia [39–44].

However, the dose required to improve hyperlipidemia in dogs is not known. Freeman et al. [45] recommended omega-3 supplementation in order to achieve a cardioprotective effect, 40mg/kg EPA and 25mg kg of DHA for both dogs and cats with cardiac disease. However, the study did not make it clear whether these dosages would be ideal for all animals with heart disease at any stage. According to Teixeira & Brunetto [31] the supplementation of omega-3 polyunsaturated fatty acids EPA and DHA, given as 5.0% of fish oil inclusion in the diet of diabetic dogs (which corresponds to an intake of EPA and DHA of 142.9mg/kg, equivalent to 253.8mg/kg^0.75^) reduces the cholesterolemia and the non-HDL cholesterol. According to Bauer [46], the recommended dose for the treatment of dogs with hyperlipidemia is 120mg EPA + DHA/kg^0.75^; however, in this recommendation, there is no references to the studies that originated the dosage. Xenoulis and Steiner [3] also suggest that the hypolipidemic effect of omega-3 is dose-dependent and, therefore, to obtain this effect, a much higher dose than that recommended so far may be necessary.

However, the dose used in the present study was slightly higher than the traditional Freeman study [45], but lower than Bauer’s recommendations [46]. That is, 1 capsule of 1000mg per animal with an average BW of 7 kg (instead of 500mg for each 7 kg of BW recommended by the manufacturer), which results in 58.8mg/kg of EPA and 45.4mg/kg of DHA, ie 104.6mg of EPA + DHA/kg, however, the results found were interesting. In our study, no side effects were reported, except for the strong fish odor mentioned by the animals’ owners, similar to that reported by Freeman [45].

Dogs belonging to Group 2 consumed more fat than Group 1, meaning that the improvement in serum TG and TC concentrations was due to the beneficial effect of omega-3 in these animals. It is worth mentioning that six of the eight animals in group 2 had mild hyperlipidemia (TG = 150 to 400mg/dL); one moderate hyperlipidemia (TG = 612mg/dL) and another severe hyperlipidemia (TG = 1.140mg/dL). These last two did not show normalization of TG values, but their concentrations were reduced by half. Therefore, in situations where TG concentrations are higher, the association with a low or ultra-low fat diet is extremely important.

Whenever possible, therapy should be combined and the severity of hyperlipidemia must be considered, in such a way that mild to moderate hyperlipidemia can be treated initially with a low-fat diet associated with omega-3 and in severe cases, bezafibrate can be associated [47].

Dogs have four LP fractions, which correspond to VLDL, LDL, HDL and HDL_1_. In hyperlipidemia, particularly in severe hypercholesterolemia, there is a substancial increase in HDL_1_ cholesterol concentration (46), unlike man, in which more than 50% of plasma cholesterol is transported by LDL. The lipid profile of healthy dogs is characterized by low concentrations of VLDL-C and LDL-C and high concentration of HDL-C [9,48].

Mori et al. [9] showed no differences in cholesterol distribution among LP particles of Schnauzers and Shetland Shephards with primary hyperlipidemia. In the current investigation, despite the reduction in cholesterolemia and triglyceridemia, the therapies did not change the distribution of cholesterol and TG among the LP fractions, which is alike to the LP profile of healthy animals [6]. It was expected that Schnauzers with primary hyperlipidemia would have higher concentrations of VLDL-TG, or the combination of VLDL and CM, with or without hypercholesterolaemia, but this result was not found.

The current investigation shows that both therapies (low-fat diet + omega-3 or moderate-fat diet + omega-3) were effective in reducing plasma TC and TG without altering LP profile.

Therefore, the lipid-lowering effect evidenced by dietary supplementation with omega-3 may have beneficial effects for Schnauzer dogs with mild to moderate primary hyperlipidaemia.

## Study limitations

The findings of this study have to be seen in light of some limitations. There were more hyperlipidemic dogs in the Group 2 (moderate-fat diet + omega-3) as compared to the Group 1 (low-fat diet + omega-3). Such evidence was due to the random and sequential distribution of animals between the experimental groups in the beginning of the study. Also, factors such as groups, sex, age, and previous diet can be confounding factors and are limits our understanding of results, but this type of standardization in clinical studies, especially with specific breed and health condition, is very difficult and do not detract from the merits of this preliminary study.

We showed that both treatments reduced hyperlipidemia. However, if there were greater availability of hyperlipidemic Schnauzer dogs in the study, we could have a control group and supposedly investigate further groups 3 (low-fat diet) and 4 (moderate-fat diet) and carry out additional comparisons among all of experimental groups which could show more clearly the lipid-lowering effects of omega-3.

As already mentioned, the results reflect the supplementation of omega-3 and dietary factors, without it being possible to isolate the responsible factors. In this sense, further studies are required to effectively show the strict physiological outcomes and dose-response effects that account for the benefits of dietary effects and omega-3 on lipid metabolism and, thus, help to engender specific therapeutic approaches using omega-3 for primary hyperlipidemic Schnauzer dogs.
